# American Medical Association

**Published:** 1852-05

**Authors:** 


### AMERICAN MEDICAL ASSOCIATION.

For the article with this caption we are indebted to John C. Darby, M. D., of Lexington. It is hardly so much a review as an original essay on various practical topics. Dr. D. promises us similar favors hereafter.

We have not received the proceedings of the last meeting of the Association, and presume we shall not, only enough copies having been preserved from the flames to supply subscribers.

The next meeting of the Association is to be held at Richmond, and will take place on the first Tuesday in this month. There is reason to expect that the meeting will be large and the proceedings interesting. Valuable reports may be looked for from the several committees. A number of delegates will be present from this city, from some of whom we hope to receive an early account of the meeting.

